# Responses of soil microbial communities and enzyme activities under nitrogen addition in fluvo-aquic and black soil of North China

**DOI:** 10.3389/fmicb.2023.1249471

**Published:** 2023-08-17

**Authors:** Sami Ullah, Muhammad Mohsin Raza, Tanveer Abbas, Xian Guan, Wei Zhou, Ping He

**Affiliations:** ^1^Ministry of Agriculture Key Laboratory of Plant Nutrition and Fertilizer, Institute of Agricultural Resources and Regional Planning, Chinese Academy of Agricultural Science, Beijing, China; ^2^ORIC, University of Baltistan, Skardu, Pakistan; ^3^Soil Environment and Chemistry Program, Land Resources Research Institute National Agriculture Research Center, Pakistan Agricultural Research Council, Islamabad, Pakistan; ^4^Mosaic Fertilizers (Beijing) Co., Ltd, Beijing, China

**Keywords:** nitrogen fertilization, soil microbial community composition, extracellular enzyme activities, fluvo-aquic soil, black soil

## Abstract

This research investigates the impact of long-term nitrogen (N) addition on fluvo-aquic and black soils in north China, with a focus on soil microbial communities and enzyme activities. In each site, there were three N fertilization treatments, i.e., control, moderate-N, and high-N. Phospholipid Fatty Acid Analysis was employed to analyze the microbial community composition, and enzyme activities related to N, carbon (C), and phosphorus (P) cycling were assessed. The results showed that increasing N fertilization levels led to higher soil organic carbon (SOC) and total N (TN) concentrations, indicating enhanced nutrient availability. N fertilization reduced soil pH across both soils, with a more pronounced acidification effect observed in the black soil. Across both soils, N addition increased maize yield, but the higher crop yield was attained in moderate-N rate compared with high-N rate. Microbial community composition analysis revealed that N fertilization induced shifts in the relative abundances of specific microbial groups. The black soil exhibited pronounced shifts in the microbial groups compared to the fluvo-aquic soil, i.e., decreased fungal abundance and fungi: bacteria ratio in response to N input. In addition, the application of N fertilizer led to an elevated ratio of gram-positive to gram-negative (GP:GN) bacteria, but this effect was observed only in black soil. N fertilization had an impact on the enzyme activities related to C, N, and P cycling in both soil types, but black soil showed more pronounced changes in enzyme activities. Permutational multivariate analysis of variance indicated that soil types rather than N fertilization mediated the response of the soil microbial community and enzyme activities. Partial least square path modeling demonstrated that soil pH was the only key driver impacting soil microbial groups and enzyme activities in both soils. In conclusion, our findings highlighted that N fertilization exerted more pronounced impacts on soil biochemical properties, microbial community composition, and enzyme activities in black soil furthermore, moderate N rate resulted in higher crop productivity over high N rate.

## Introduction

China’s agricultural industry is a significant contributor to global food production, boasting a cultivation area that accounts for less than 9% of the world’s total but sustains almost 20% of the world’s population (Liu et al., 2021). The remarkable success in agricultural productivity in China can be attributed to the widespread utilization of chemical fertilizers (Ding et al., 2018). North-central China is predominantly occupied by ‘fluvo-aquic soil’ and north-east China is dominated by ‘black soil.’ Both regions hold considerable significance for China’s food security as they contribute to the production of more than 22% of the country’s total grain output (Gong et al., 2009; Xu et al., 2016). In recent decades, the pursuit of increased agricultural productivity has led to the widespread adoption of intensive fertilization practices in two specific regions of China. However, this excessive use of fertilizers has resulted in significant degradation of soil quality and adverse environmental consequences (Cui et al., 2008; Xu et al., 2016). Therefore, the need for a significant transition from higher N rates to optimum or moderate rates for sustainable agricultural development has become increasingly apparent in recent years. This shift is crucial in order to and safeguard the ecological environment and ensure sustainable food security (Davies and Shen, 2020).

Soil microorganisms have a pronounced impact on the processes of soil organic matter (SOM) formation and decomposition, maintaining productivity, nutrient cycling, and carbon sequestration (Song et al., 2020). The soil microbial population’s role extends to regulating crop production and suppressing diseases (Van Der Heijden et al., 2008; Berg, 2009). Despite their small proportion within the overall soil composition, microorganisms are integral to the cycling of key elements such as carbon (C), nitrogen (N), phosphorus (P), and sulfur (S). Moreover, soil microbes play a significant role in maintaining soil fertility and quality (Wei et al., 2019; Song et al., 2020). However, it has been found that unplanned and non-scientific anthropogenic activities, such as fertilization and tillage, exert a notable influence on soil microbial communities (Ding et al., 2017), consequently hampering soil ecosystem functioning.

The impact of N fertilizer application on microbial taxa involved in soil nutrient cycling has been well-established (Fierer et al., 2012; Ding et al., 2017). Nevertheless, modifications in soil properties exhibit a strong correlation with changes in microbial community structure (Song et al., 2020). Earlier research has highlighted soil pH as a crucial environmental factor influencing microbial community structure (Dai et al., 2019; Song et al., 2020). Furthermore, long-term N fertilization-induced alterations in soil carbon and C:N ratios can also impact soil microbial community structures (Dai et al., 2019). Apart from soil properties, earlier studies have shown that soil microbial populations can be influenced by some other factors, such as tillage system (Hartmann et al., 2015), crop species (Li et al., 2021), soil type (Yu et al., 2019), and fertilizer regimes (Carey et al., 2015; Su et al., 2015; Wang et al., 2018). Likewise, soil extracellular enzyme activities (EEA) are impacted by soil properties and fertilizer regimes (Wang et al., 2018; Song et al., 2020).

Soil microorganisms exhibit a remarkable ability to decompose SOM through various EEA, which play a crucial role in breaking down complex compounds (Whitaker et al., 2014). In other words, all biochemical changes in soil depend on enzyme reactions. Moreover, soil enzyme activity reflects the magnitude and direction of numerous biochemical processes. Consequently, soil EEA is regarded as an essential indicator of soil fertility and soil quality (Dick, 1997). Although application of inorganic fertilizers has been shown to increase enzyme activities (Ai et al., 2012) the effects of chemical N in different soil types on enzyme activities needs further studies. In addition, the interactions between soil biology, physics, chemistry, and EEA in different soil types have not yet been investigated (Burns et al., 2013).

Therefore, to provide a comprehensive understanding of the N application-induced shifts in soil microbial communities and EEA and their interactions with soil variables, we sampled soil from two long-term field experiments, i.e., fluvo-aquic soil of northcentral China and black soil of northeast China. The present study investigates the impact of fertilization regimes, soil types, and soil properties on soil microbial composition and extracellular enzyme activities associated with C, N, and P acquisition in long-term field experiments with the following objectives: (i) to observe shifts in soil microbial groups and enzyme activities in response to N fertilization; (ii) to identify the impact of N fertilization versus soil types on soil enzymes and microbial communities; and (iii) to identify important soil variables influencing soil enzyme activities and microbial communities. We hypothesize that the application of N fertilization will lead to significant shifts in soil microbial communities and enzyme activities. Both nitrogen fertilization and soil types will have a substantial impact on soil enzymes and microbial communities, resulting in variations in response across different soil types. Through our study, we will identify important soil variables that significantly influence soil enzyme activities and microbial communities, helping us understand the key factors driving these ecological processes.

## Materials and methods

### Site description

A long-term field trial was established in 2009 at the Dahe Experimental Station, located in Shijiazhuang City, Hebei Province, North Central China (38°07′ N and 114°29′ E). This region is characterized by fluvoaquic soil and exhibits a typical temperate and subhumid continental monsoon climate, with an average annual temperature of 14.3°C and an annual precipitation of 400 mm. Another field trial was also initiated in 2009 in Liufangzi County, Gongzhuling City, Jilin Province, situated in northeastern China (43° 34.86 N and 124° 53.92 E). The study area in Liufangzi County features black soils known as haplic phaeozems (FAO classification) and mollisols (USDA classification). It experiences a temperate and semi-humid continental monsoon climate, with an average annual temperature ranging from 4 to 5°C and an annual precipitation between 500 and 900 mm.

### Experimental design

The experimental design employed in both locations was a randomized complete block with four replicates. Each plot size in the fluvo-aquic soil site was 45 m^2^ (5 × 9 m), while in the black soil site, it was 40 m^2^ (10 × 4 m). In fluvo-aquic soil, three treatments were implemented, namely the control, moderate-N182 kg ha^−1^, and high-N225 kg ha^−1^. Similarly, in black soils, three treatments were implemented, including a control, moderate-N200 kg ha^−1^, and high-N251 kg ha^−1^. A total of 24 experimental plots were used, resulting from combining 3 treatments, 4 replications, and 2 sites. In both sites, the moderate-N treatments represented lower N fertilization compared to the general farmer’s fertilization rate. On the other hand, the high-N treatments represented the typical fertilization rate used by farmers in north-central (fluvo-aquic soil) and northeast (black soil) China. In treatments, all the N, P, and potassium (K) were applied as a basal application. Urea was used as the source of N fertilizer in this study, and no organic fertilizer was used. Other agricultural practices, such as tillage and pesticide application, remained the same for both the moderate-N fertilization and high-N fertilization treatments. In north-central China, the crop rotation system consisted of winter wheat followed by summer maize, while in northeast China, a summer maize monocropping system was practiced. In September 2018, we sampled soil from both study sites. Typically, five soil cores with a diameter of 2 cm were taken from each experimental plot, reaching a depth of 0–20 cm. These cores were combined to form a composite sample. A portion of the composite sample was preserved at a temperature of −80°C for subsequent molecular analysis.

### Determination of soil biochemical properties

The concentrations of soil organic carbon (SOC) and total nitrogen (TN) were determined through dichromate oxidation (Kalembasa and Jenkinson, 1973) and Kjeldahl digestion (Bottomley et al., 2020), respectively. The concentration of soil inorganic N, specifically NO_3_^−^N and NH_4_^+^N, was determined by analyzing a 12 g fresh soil sample mixed with a 0.01 mol L^–1^ CaCl_2_ solution at a 1:10 ratio. The analysis was conducted using continuous flow analysis with the Foss FlAstar 5,000 instrument from Sweden. The pH of the soil was determined by employing a compound electrode (PE-10, Sartorius, Germany) and utilizing a soil-to-water ratio of 1:2.5. The fumigation extraction method using 0.5 M K_2_SO_4_ was employed to conduct the microbial biomass C (MBC) and microbial biomass N (MBN) analyses (Brookes et al., 1985), and the results were determined using a total organic C/N analyzer (Multi N/C 3100/HT1300, Analytik Jena AG).

### Determination of soil extracellular enzyme activities

Comprehensive details regarding the EEA that are associated with P, N and C cycling are presented in Table 1. The fluorescence-based protocols as described by Ai et al. (2012) were utilized to determine the activities of extracellular enzymes associated with nitrogen and carbon. The units of measurement used were expressed as nmol h^−1^ g^−1^. In summary, a quantity of 1 gram of freshly collected soil was subjected to homogenization in 100 milliliters of sterilized water with the aid of a polytron homogenizer. Subsequently, a magnetic stirrer was employed to ensure the homogeneity of the suspension. The experimental procedure involved the introduction of a suspension sample, sterilized water, 200 μM of 4-methylumbelliferyl-linked substrates, and 10 μM of references into the wells of a black 96-well microplate. The microplates were incubated in darkness at a temperature of 25°C for a duration of 4 h while being covered. Following the incubation period, a 10 μl aliquot of a 1 M NaOH solution was promptly introduced into every well of the microplate to stop the enzymatic reaction. The quantification of fluorescence was conducted utilizing a microplate fluorometer (Scientific Fluoroskan Ascent FL, Thermo Fisher Scientific, Waltham, MA, USA), with the implementation of 365 nm excitation and 450 nm emission filters.

**Table 1:** Phospholipid fatty acids used as signature biomarkers.

Microbial groups						
General bacteria	Gram + bacteria	Gram-bacteria	Fungi	Arbuscular mycorrhizal fungi	Saprophytic fungi	Actinomycetes
**PLFA biomarkers**						
14:0, 15:0, 16:0, 17:0	i14:0, i15:0, i16:0, i17:0, i18:0	16:1 w7c, 17:1 w8c, 18:1 w7c, 18:1 w5c	18:1 w9c	16:1 w5c	18:2 w6c	16:0 (10Me), 17:0 (10Me), 18:0 (10Me)

### Phospholipid fatty acid analysis

The soil microbial community and microbial biomass were assessed using phospholipid fatty acid analysis (PLFA), following the method described by Wu et al. (2009). In brief, 3 g of freeze-dried soil samples were utilized for PLFA extraction. A chloroform/methanol/citric acid buffer (volume ratio of 1:2:0.8, pH 4.0) was used for the extraction process. The polar lipids were separated from glycolipids and neutral lipids by eluting with acetone and chloroform, respectively, on a silica-bonded phase column. To serve as an internal standard, nonadecanoic acid methy ester (19:0) was added. The polar lipids were further converted to fatty acid methyl esters (FAMEs) through mild alkaline methanolysis. The resulting dried FAMEs were dissolved in n-hexane and subsequently analyzed and identified using the MIDI Sherlock microbial identification system version 4.5 (MIDI Inc., Newark, DE, USA) and gas chromatography (Agilent N6890). The concentrations of PLFAs were expressed as nmol g^−1^ of dry soil. The total microbial biomass was determined by calculating the sum of all PLFA concentrations (nmol g^−1^), while the relative abundance of each PLFA was determined by its percentage mole abundance in each sample. Various taxonomic groups of microorganisms were identified using specific PLFAs as biomarkers, including bacteria, gram-positive (G+) and gram-negative (G–) bacteria, fungi, arbuscular mycorrhizal fungi (AMF), saprophytic fungi, and actinomycetes. Table 2 shows the PLFAs that were used as signatures for each taxonomic group of microorganisms based on fatty acid biomarker data that has already been published (Ai et al., 2012; Willers et al., 2015; Dai et al., 2019).

**Table 2:** Extracellular enzymes assayed and their enzyme commission number (EC).

Nutrient cycle	Enzyme	Abbreviation	Substrate	EC
N cycle	α-glucosidase	AG	4-MUB-α-_D_-glucoside	3.2.1.20
	l-leucine aminopeptidase	LAP	_L_-Leucine-7-amino-4-methylcoumarin	3.4.11.1
	*N*-acetyl-*β*-glucosaminidase	NAG	4-MUB-*N*-acetyl-*β*-_D_-glucosaminide	3.2.1.30
C cycle	*β*-glucosidase	BG	4-MUB-*β*-_D_-glucoside	3.2.1.21
	*β*-cellobiosidase	BC	4-MUB-*β*-_D_-cellobioside	3.2.1.91
	β-xylosidase	BX	4-MUB-β-_D_-xyloside	3.2.1.37
P cycle	Phosphatase	PHOS	4-MUB-phosphate	3.1.3.1

### Data analyses

To assess the significance of each measured variable, such as soil chemical properties, microbial populations, and enzyme activities a one-way analysis of variance (ANOVA) was conducted. Fisher’s least significant difference (LSD) test was used to compare the means of different treatments, with a significance level set at *p* ≤ 0.05. The statistical analysis was carried out using SPSS version 24. To differentiate the individual contributions of soil types and N fertilization on soil enzyme activities and microbial populations, a permutational multivariate analysis of variance (PERMANOVA) was performed using Primer software version 6.0. We utilized principal component analysis (PCA) to detect alterations in enzyme activities and microbial community structure following nitrogen (N) addition in two distinct soil types using Conoco 5. To examine the interrelationships between soil properties, enzyme activities, and microbial community composition, we employed a statistical technique called partial least squares path modeling (PLSPM). This approach allows us to visualize and analyze the cause-and-effect relationships among observed and latent variables. In PLSPM, path coefficients represent the direct effects, indicating the direction and strength of the linear relationship between variables. Additionally, indirect effects are considered, which involve the multiplied path coefficients between a predictor and a response variable, considering all possible paths except the direct effects. To validate the estimates of the coefficients of determination (*R*^2^) and the path coefficients in our model, we utilized the “plspm” package in R software version 3.4.4. The validation process involved performing 1,000 bootstraps, which helped assess the stability and reliability of the model estimates.

## Results

### Soil biochemical properties and maize yield

The impact of N fertilization on soil chemical properties and maize yield was investigated in two different soil types: fluvo-aquic soil and black soil (Table 3). In the fluvo-aquic soil, pH values did not show significant differences among the treatments. However, in the black soil, both moderate-N and high-N treatments resulted in a significant decrease in pH compared to the control. Total N content was significantly higher in the fluvo-aquic soil with both moderate-N and high-N treatments. In the black soil, nitrate nitrogen (NO_3_^−^N) levels were significantly increased by both moderate-N and high-N treatments, while Total N, SOC, and MBC content showed slight differences among the treatments. MBN content was significantly impacted by N application in fluvo-aquic soil but not in black soil. Regarding maize yield, the moderate-N treatment resulted in the highest yield in both soil types, followed by the high-N treatment. However, the crop yield was more responsive to N addition in black soil.

**Table 3:** Soil biochemical properties and maize yield under N addition in fluvo-aquic and black soil.

	pH	TN (g kg^−1^)	NO_3_^−^N (mg kg^−1^)	NH_4_^+^N (mg kg^−1^)	SOC (g kg^−1^)	C:N	MBN (mg kg^−1^)	MBC (mg kg^−1^)	Yield (kg ha^−1^)
**Fluvo-aquic soil**									
CK	7.88	1.15 b	3.07 b	1.54	18.90 b	16.44	19.63 b	208.88	5,310
Moderate-N182	7.79	1.24 a	14.65 a	1.79	20.28 a	16.39	33.63 a	271.47	6,574
High-N225	7.80	1.27 a	14.88 a	1.29	20.73 ab	16.34	26.32 ab	256.78	5,933
**One-way ANOVA *p*-values**									
P	0.07	≤0.01	≤0.001	0.55	≤0.05	0.93	≤0.05	0.11	0.08
**Black soil**									
CK	6.01 a	1.24	3.02 b	1.97 b	24.47	19.76	44.76	45.02	1905 c
Moderate-N200	5.87 b	1.27	3.85 b	2.52 b	26.66	20.94	49.32	34.82	12,040 a
High-N251	5.19 b	1.30	19.55 a	11.59 a	25.58	19.77	55.73	23.73	10,739 b
**One-way ANOVA *p*-values**									
P	≤0.001	0.4	≤0.05	≤0.001	0.29	0.58	0.13	0.61	≤0.001

The data are the means; *n* = 4. Different letters in a column indicate significant differences among treatments at *P* ≤ 0.05 probability level as determined by Fisher’s least significant difference (LSD).

### Soil microbial community composition

**Figure 1:**
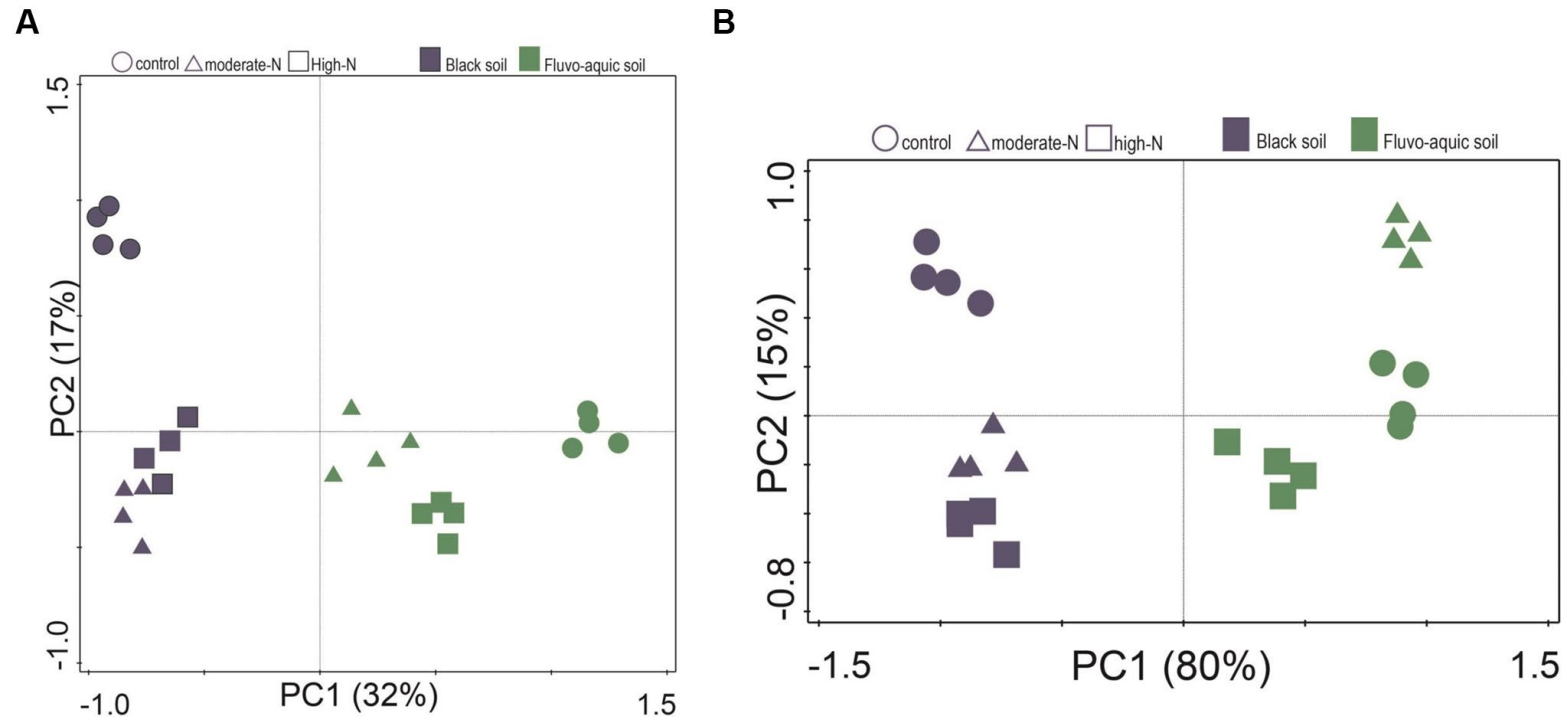
Principle component analysis (PCA) of the soil microbial community composition **(A)** and extracellular enzyme activities **(B)** data for the control, moderate-N, and high-N treatments for fluvo-aquic and black soil.

**Figure 2:**
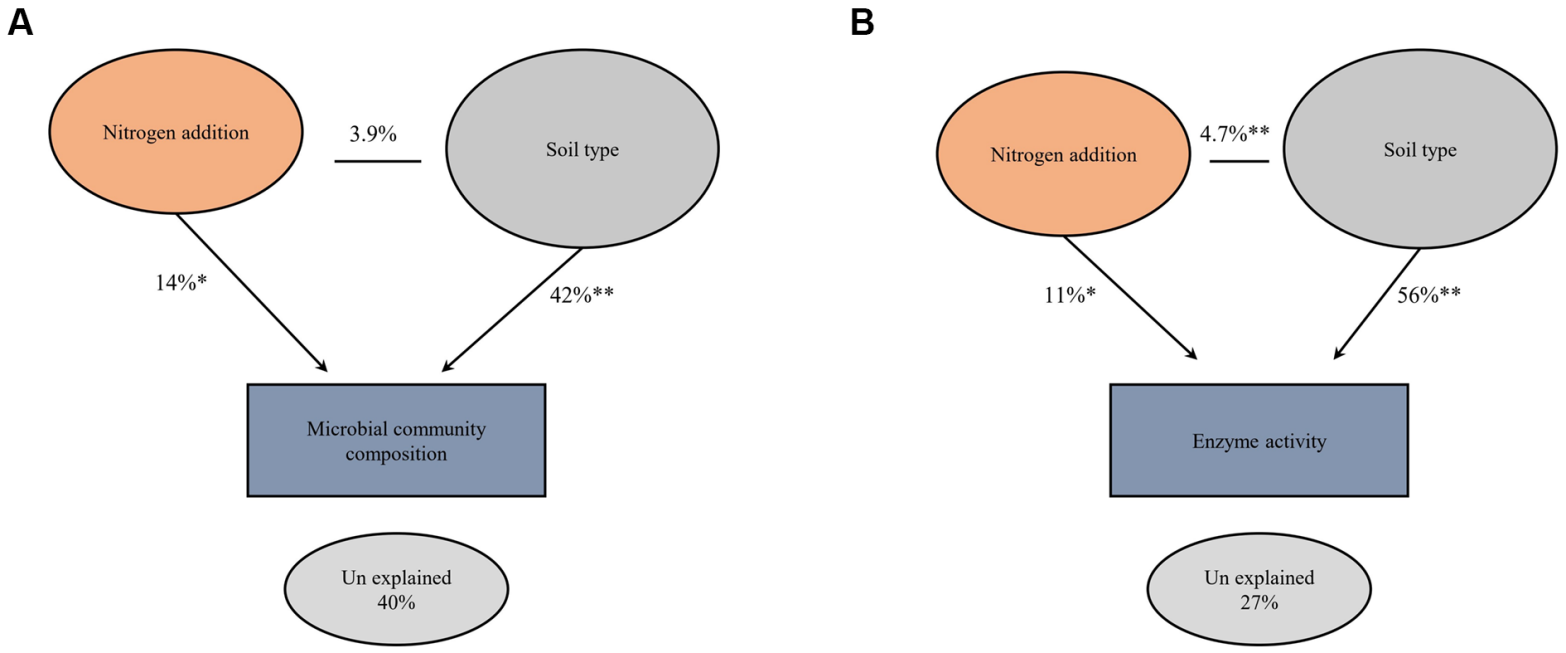
Permutational multivariate analysis of variance (PERMANOVA) comparing the main and interactive effects of N fertilizer and soil type on the soil microbial community composition **(A)** and extracellular enzyme activities **(B)** at (999 permutations). Asterisks indicate significant differences at *p ≤ 0.01 and **p ≤ 0.001 probability levels.

The impact of nitrogen fertilization on soil microbial communities was assessed in both fluvo-aquic and black soils (Table 4). In the fluvo-aquic soil, there were no significant differences in the abundance of bacteria, fungi, saprophytic fungi, and actinomycetes among the control, moderate-N, and high-N treatments. However, the abundance of AMF showed a significant decrease with an increasing nitrogen fertilization rate. Similarly, in the black soil, the abundance of bacteria did not significantly differ among the control, moderate-N, and high-N treatments. However, the abundance of fungi and AMF significantly decreased with increasing nitrogen fertilization rates. The ratio of Fungi:bacteria (F:B) decreased while the GP:GN ratio increased with N fertilization. PCA was utilized to evaluate the distinct differentiation of microbial community composition across two soil types. PCA demonstrated a clear separation of microbial communities in both fluvo-aquic and black soil (Figure 1A). To separate the individual impacts of N fertilization and soil types on microbial community composition, we employed PERMANOVA. The outcomes of PERMANOVA revealed that soil type accounted for most of the variation, specifically 42%, in microbial community composition (Figure 2A). Conversely, N fertilization contributed 14% of the variation.

**Table 4:** Relative abundance of the individual PLFAs (mol %) in soil samples from the fluvo-aquic and black soil.

	Bacteria	Fungi	SF	AMF	Actino	GP	GN	GP:GN	F:B
**Fluvo-aquic soil**									
Control	52.74	7.47	4.08	3.80 a	11.64	17.56	17.42	1.01	0.14
Moderate-N182	53.60	7.03	3.64	3.42 b	11.67	16.95	17.50	0.97	0.13
High-N225	53.48	5.65	2.9	3.07 c	12.24	18.05	17.06	1.06	0.10
**One-way ANOVA *p*-values**									
P	0.5	0.26	0.14	≤0.01	0.06	0.73	0.56	0.51	0.200
**Black soil**									
Control	47.15	5.92 a	2.26	2.97 a	9.92 a	17.57 b	13.10 a	1.34 c	0.126 a
Moderate-N200	46.72	5.65 a	2.09	2.59 b	9.91 a	18.21 b	12.17 a	1.50 b	0.121 b
High-N251	46.67	4.42 b	2.32	1.74 c	8.49 b	19.19 a	10.01 b	1.92 a	0.095c
**One-way ANOVA *p*-values**									
P	0.83	≤0.001	0.8	≤0.001	≤0.001	≤0.01	≤0.001	≤0.0001	≤0.001

Data are the mean, *n* = 4. Different letters indicate significant differences among treatments at *P* ≤ 0.05 probability level as determined by Fisher’s least significant difference (LSD). SF = saprophytic fungi, AMF = Arbuscular mycorrhizal fungi, Actino = Actinomycetes, GP = Gram positive bacteria, GN = Gram negative bacteria, F:B = Fungi:Bactrtia ratio.

### Soil extracellular enzyme activities

**Figure 3:**
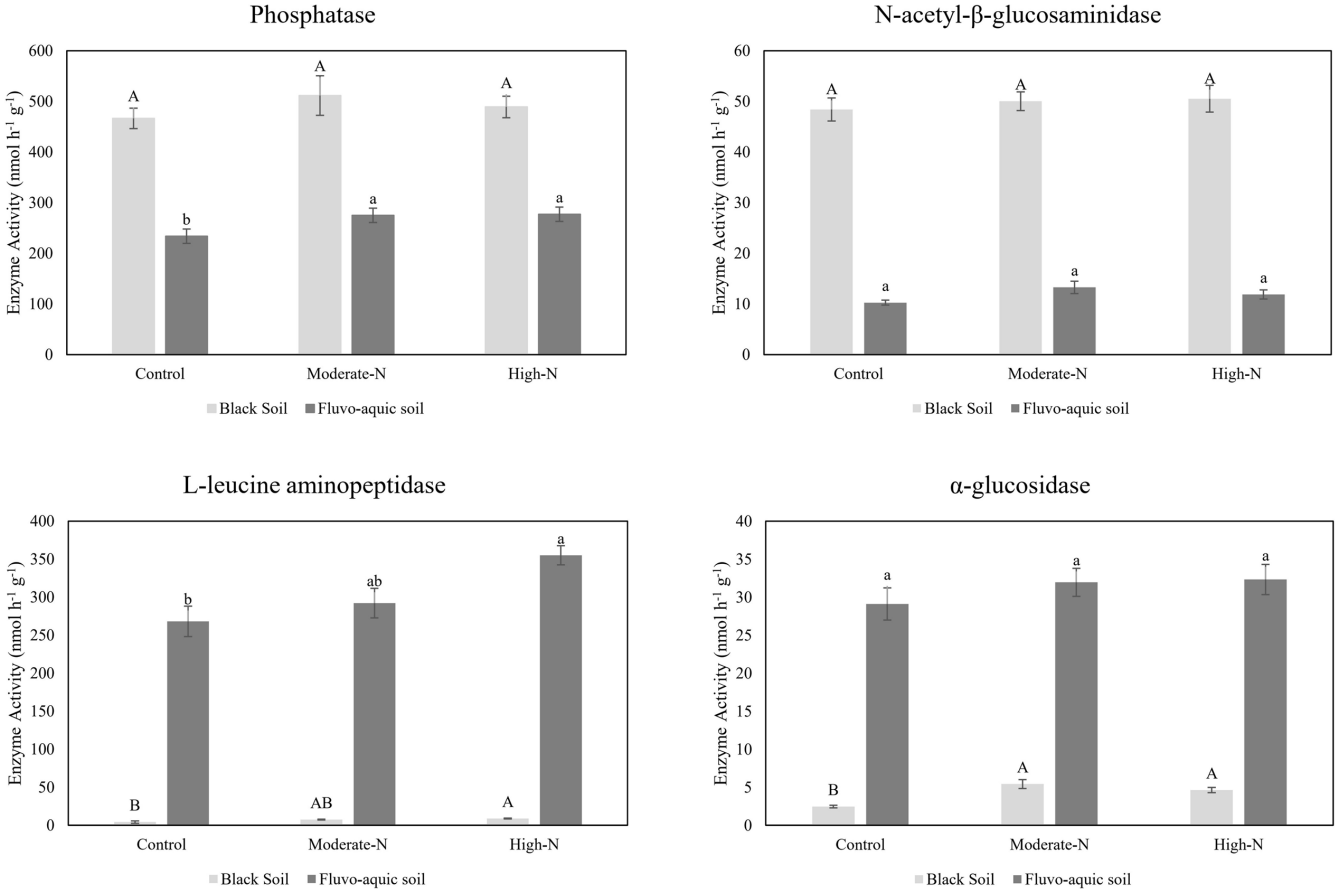
The data are the means; *n* = 4. Significantly altered N and P cycling extracellular enzyme activities in fluvo-aquic and black soil. Different letters indicate significant differences among treatments at *p* ≤0.05 probability level as determined by Fisher’s least significant difference (LSD). Note: Capital letters indicate significance in black soil while small letters indicate significance in fluvo-aquic soil.

The impact of nitrogen fertilization on extracellular enzyme activities was assessed in two soil types. The results suggest that nitrogen fertilization did not have a significant impact on soil enzyme activities except Phosphatase and LAP in fluvo-aquic soil. In contrast, in the black soil, significant differences were observed in several enzyme activities with nitrogen fertilization (Figures 3 4; Table 5); for example, BG, BX, and BC activities were significantly impacted by N application. Phosphatase and NAG activities did not show significant differences among the treatments. PCA was utilized to evaluate the distinct changes in EEA across the two soil types. PCA demonstrated clear separation of EEA in both fluvo-aquic soil and black soil (Figure 1B). The results of PERMANOVA revealed that soil type accounted for the majority of the variation, specifically 56%, in EEA (Figure 2B). Conversely, N fertilization contributed 11% of the variation. Overall, the results indicate that the influence of nitrogen fertilization on soil enzyme activities varied depending on the soil type.

**Figure 4:**
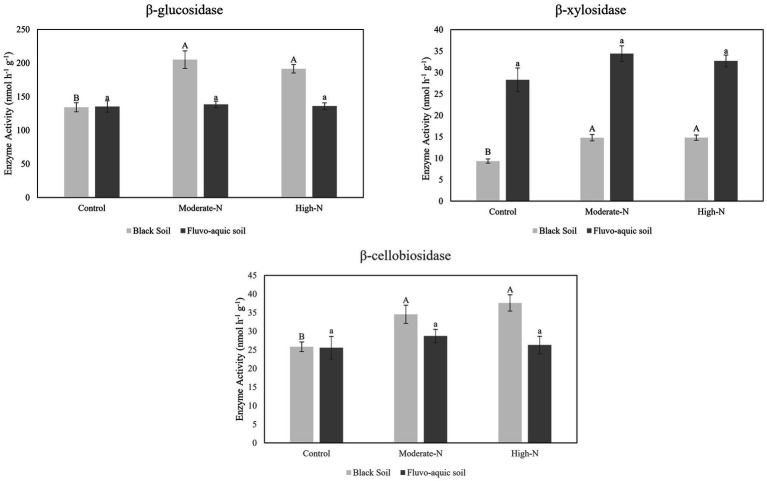
The data are the means; *n* = 4. Significantly altered C cycling extracellular enzyme activities in fluvo-aquic and black soil. Different letters indicate significant differences among treatments at P ≤ 0.05 probability level as determined by Fisher’s least significant difference (LSD). Note: Capital letters indicate significance in black soil while small letters indicate significance in fluvo-aquic soil.

**Table 5:** *P-*values derived from one-way ANOVA for extracellular enzyme activities in soil samples from the fluvo-aquic and black soil.

	PHOS	NAG	LAP	AG	BG	BX	BC
Fluvo-aquic soil	≤0.001	0.14	≤0.05	0.53	0.91	0.18	0.65
Black soil	0.66	0.84	≤0.05	≤0.001	≤0.001	≤0.001	≤0.05

For interpretation of abbreviations please refer to Table 2.

### Partial least square path modeling

**Figure 5:**
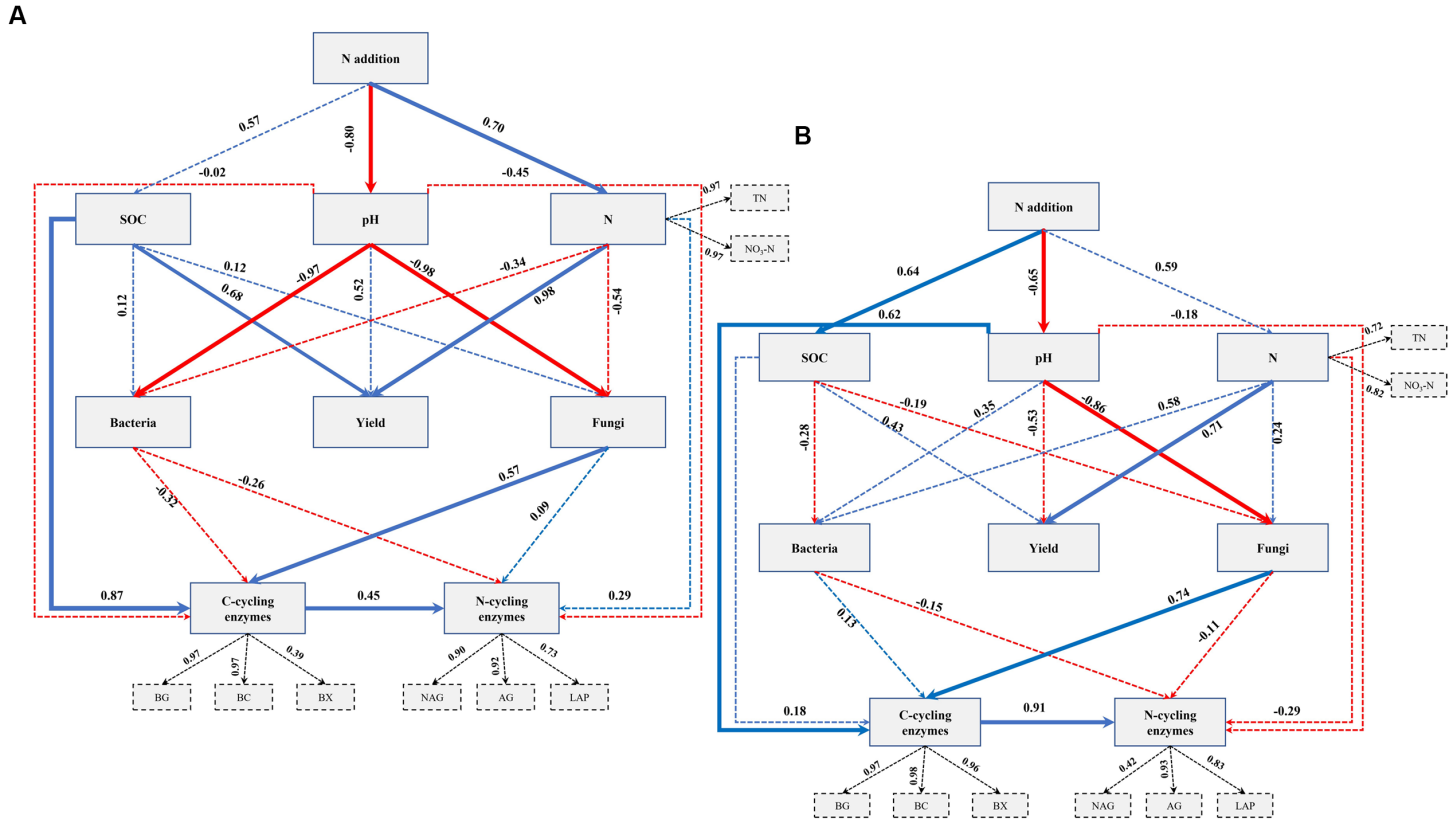
Directed graph of the partial least squares path model (PLS-PM) for fluvo-aquic soil **(A)** and black soil **(B)**. Each box represents an observed variable (i.e., measured) or latent variable (i.e., constructs). The loading for N contents and the six enzyme activities that create the latent variables are shown in the dashed rectangle. Path coefficients are calculated after 1,000 bootstraps and reflected in the width of the arrow, with blue and red indicating positive and negative effects, respectively. Dashed arrows show that coefficients did not differ significantly from 0 (*p* ≥ 0.05). The model is assessed using the Goodness of Fit (GoF) statistic, and the GoF values were 0.70 and 0.66 in the fluvo-aquic and black soil, respectively.

To investigate the relationships between N fertilization, maize yield, soil properties, soil bacterial and fungal communities, and EEA, a PLSPM analysis was conducted. The findings in fluvo-aquic soils revealed that the addition of N exhibited a significant direct relationship with soil N contents and pH (Figure 5A). Moreover, soil pH significantly influenced both fungal and bacterial communities. It was observed that soil N contents had a significant direct association with crop yield. Additionally, SOC and soil fungi exhibited a significant relationship with C-cycling enzymes, while no significant relationship was found between soil bacteria and C-cycling enzymes. Notably, C-cycling enzymes displayed a significant direct relationship with N-cycling enzymes. In the context of black soil, the N application exhibited a significant direct association with soil pH and SOC (Figure 5B). Soil N contents exerted a significant influence on crop yield. Furthermore, soil pH demonstrated a significant direct correlation with soil fungal communities and EEA involved in C cycling, whereas no significant relationship was observed with soil bacterial communities. Importantly, a significant direct relationship was observed between C-cycling enzymes and N-cycling enzymes.

## Discussion

### Nitrogen addition impacts on soil biochemical properties and maize yield

The application of N fertilizer impacted soil chemical properties and increased nutrient contents across both soil types (Table 3). Earlier research has indicated that extended application of nitrogen fertilizers resulted in an enhanced level of SOC, overall N content, and particulate organic matter (Dai et al., 2019; Yu et al., 2019). It has been established that N fertilization can directly affect soil physiochemical properties, which in turn can have a significant impact on crop production. In particular, PLSPM indicated that the soil chemical properties were influenced by the addition of N, which in turn had a direct or indirect effect on the yield of maize. The levels of available N, TN, and SOC were identified as significant factors in regulating maize grain yield, either through individual or interactive effects (Figure 5). A study by Yu et al. (2019), found that SOC played a significant role in enhancing crop yields through mediating microbial biomass and soil enzyme activities. The findings of Luo et al. (2018) in a comprehensive meta-analysis indicate that N fertilization can enhance crop production by augmenting SOC and TN levels, as well as biological soil enzyme activity across diverse cropping systems worldwide.

The N fertilization induced changes can have an impact on the soil microbial community, leading to significant modifications in soil microbial characteristics such as total microbial biomass, MBC, and MBN contents. In the present study, divergent responses of MBC were observed in the two soils (Table 2). The divergent responses of MBC to N fertilization in the two soils could be related to differences in their initial microbial communities and nutrient utilization strategies (Sun et al., 2021). Some microbial groups are specialized in efficiently utilizing N and other nutrients, while others may rely on different carbon sources (Xu et al., 2021). Additionally, the initial nutrient status of the soils can also influence microbial responses to N fertilization. If one soil already has a relatively high nutrient content, including nitrogen, the additional nitrogen from the fertilization might not have as pronounced an effect on microbial growth compared to a soil with low initial nutrient levels (Chen et al., 2020). It has been established that the application of N fertilizer results in a significant increase in soil MBN and MBC contents, regardless of soil type (Yu et al., 2019). Conversely, in a meta-analysis conducted by Jian et al. (2016), it was found that the application of N fertilization resulted in a decrease in MBC content. Results in Table 3 showed that the application of N fertilization had a greater impact on reducing the pH of black soil compared to fluvo-aquic soil. One potential reason for the greater impact of N fertilization on reducing the pH of black soil compared to fluvo-aquic soil could be related to differences in the buffering capacity of the two soil types. Buffering capacity refers to a soil’s ability to resist changes in pH when acidic or alkaline substances are added. Soils with higher buffering capacity can maintain a more stable pH even when external factors, such as fertilizer application, attempt to alter it (Lopes et al., 2015). In addition, distinct soil types exhibit distinct initial characteristics (Staley et al., 2018; Yu et al., 2019). Consequently, the impact of N fertilization on different soil properties was found to be diverse (Yu et al., 2019).

In the present study, the higher N rate resulted in substantial soil acidification in fluvo-aquic soil and black soil. The application of additional N treatments resulted in a reduction in pH levels, irrespective of the soil type, which consequently led to soil acidification (Guo et al., 2010). The phenomenon of soil acidification has been observed to have significant impacts on the availability of nutrients for plants and the decomposition of soil organic matter (Treseder, 2008). In order to ensure the preservation of soil quality and mitigate the environmental consequences associated with agriculture, it is imperative to prioritize the reduction of fertilizer rates, specifically N fertilizer. This approach is crucial for maintaining optimal crop productivity in a sustainable manner. In pursuit of this objective, the Chinese government has implemented a comprehensive initiative known as the Zero Growth of Chemical Fertilizer Use by 2020 (Shuqin and Fang, 2018). The implementation of such initiatives is anticipated to have multiple benefits, including the maintenance of elevated crop productivity levels, improved efficiency in nutrient utilization, and the safeguarding of the environment against the detrimental impacts associated with agricultural practices.

### Soil microbial community composition in response to nitrogen addition

The potential impact of N enrichment on soil ecosystem stability is a topic of concern in ecological research. It is well established that sustaining of soil productivity and ecological equilibrium in agroecosystems is heavily reliant on the abundance and diversity of soil microorganisms (Zhou et al., 2017). Specifically, the reduction in abundance and diversity resulting from N enrichment may have negative consequences for the interactions between above- and below-ground ecosystems (Tilman et al., 2006; Zhou et al., 2017).

In the present study significant reduction of fungal abundance in response to N addition was witnessed in black soil while no significant shifts were observed in fluvo-aquic soils. The specific shifts in fungal microbial community composition are attributed to ecosystem N status, where N additions have little effect the abundance of fungal communities in fluvo-aquic soil (Wang et al., 2018). The response of fungal microbial communities to N addition can also be influenced by changes in soil pH. N fertilizers can lead to increased soil acidity due to the nitrification process. Fungi generally prefer slightly acidic to neutral pH conditions, and the reduction of pH in black soil might have negatively impacted fungal growth, contributing to the observed decline in their abundance. In addition, Fungi and bacteria often compete for limited resources in the soil. Nitrogen addition could have intensified this competition, affecting fungal communities more severely in black (Bluhm et al., 2019).

Despite of earlier reports that bacterial growth is more susceptible to low pH compared to fungi (Pennanen et al., 1999 Rousk et al., 2010), our observations did not reveal a significant reduction in bacterial abundance or increase in F:B ratio in plots with lower pH levels (Table 4). The findings of our study indicated the possibility that certain bacterial groups have developed significant adaptations to environmental stressors induced by nitrogen in the study area. Our results revealed that N input consistently decreased F:B ratio across both the soils but particularly in black soil. The study conducted by Strickland and Rousk (2010) found that the observed responses between the F:B and microbial C:N to N addition were primarily due to the higher biomass C:N of the fungal communities compared to the bacterial communities. This result holds significant implications. Firstly, the microbial C:N ratio can serve as an indicator to determine the relative abundance of fungi over bacteria in a given environment (Nannipieri et al., 2003). Secondly, extracellular enzymes serve as the immediate agents for the decomposition of soil organic matter. These enzymes’ activities can serve as indicators for soil nutrient cycling, and microbial soil respiration (Jian et al., 2016). Therefore, the reduction in microbial biomass does not necessarily imply that the addition of N prevents the growth of microorganisms (Zhou et al., 2017).

The application of N fertilizer resulted in a shift in the GP:GN ratio of bacteria (Table 4). Schimel et al. (2007) demonstrated that within the domain of microorganisms, GP bacteria possess a strong and intricately connected peptidoglycan cell wall, whereas GN bacteria exhibit a single-layer cell wall and an outer membrane. However, earlier research has identified ecological and physiological differences between GP and GN bacteria. The predominant microorganisms that facilitate the decomposition of litter and soil organic matter are GP bacteria and fungi. A significant number of microorganisms that perform specific or limited functions in soil are Gram-negative (GN) bacteria (Schimel, 1995). According to earlier research, it is believed that GP bacteria possess a greater inherent resistance to environmental stress when compared to GN bacteria (Schimel, 1995; Schimel et al., 2007). The observed increase in GP:GN appears to be consistent with the increase in soil available N resulting from the N application, as indicated in Table 4. The study conducted by Fanin et al. (2013) highlights the significance of P-driven stoichiometric control in understanding the spatiotemporal heterogeneity of the GP:GN. In particular, the positive response of the GP:GN ratio has been observed to potentially facilitate the breakdown of recalcitrant SOM and enhance C acquisition by soil microbiota (Zhou et al., 2017).

### Soil enzyme activities in response to nitrogen addition

Soil EEA serves as significant biological indicators of soil biodiversity, ecosystem function, and soil fertility levels by reflecting nutrient cycling activities (Sinsabaugh et al., 2009; Wallenstein and Burns, 2011). We observed that glycosidases activities (*β*-cellobiosidase, *β*-glucosidase, *β*-xylosidase, and *α*-glucosidase) were enhanced following the addition of N (Figures 3, 4). A meta-analysis conducted on the effects of N additions on glycosidase activities across various ecosystems showed that anthropogenic N input stimulated the activities of glycosidases (Jian et al., 2016). In present study, N fertilization plots had enhanced activities of Phosphatase. The production of AP requires elevated levels of N, as noted by Treseder and Vitousek (2001). Consequently, the application of nitrogen amendments may have potentially stimulated microbial activity, resulting in increased AP production. Furthermore, the acidification of soil induced by N application could potentially contribute to the mineral soil’s phosphorus binding capacity and subsequently decrease the accessibility of phosphorus to microorganisms. In order to fulfill the necessary nutrient requirements for phosphorus, microorganisms have the ability to synthesize a greater quantity of phosphatase (Sinsabaugh et al., 2009). We further observed that N-treatment plots exhibited an increase in specific enzyme activities while some enzymes activities stay neutral. This suggests that microbial populations carried out a more pronounced enzyme activity in N treated plots (Francioli et al., 2016). Moreover, it is has been observed that specific extracellular enzyme activities are better indicator of the metabolic status of a microbial community in acquiring C and nutrients, as compared to absolute enzyme activities which only reflect gross microbial activity (Raiesi and Beheshti, 2014).

### Impact of nitrogen fertilization vs. soil type on microbial community composition and enzyme activities

The findings of our study indicated that the microbial community structure and enzyme activities of soil were primarily influenced by soil type, rather than N fertilization (Figures 2A, B). Earlier research claimed that the primary factor influencing the significant variation in the microbial community composition and enzyme activities on a large scale is geographic location/soil type rather than fertilization (Zhang et al., 2021). Additionally, it has been suggested that environmental variables resulting from N fertilization may have a lesser impact on this variation (Chen et al., 2016). Based on soil sampled across 03 different soil types in China, Zhang et al. (2021), reported that geographical distance explained 61.60% of the variance in microbial community composition, but N fertilization only accounted for 1.52%.

### Exploring impacts of N fertilization-driven soil variables with microbial communities and enzyme activities

Nitrogen fertilization impacts soil microbial activity, nitrogen, and carbon dynamics, indicating that soil microbial communities can regulate nitrogen-use efficiency and carbon dynamics efficiency to mitigate resource imbalances (Mooshammer et al., 2014). The maintenance of ecosystem sustainability and productivity is heavily influenced by the presence of a suitable community structure and ample diversity of soil microbes, which play a crucial role in regulating various soil biogeochemical processes (Schimel, 1995; Strickland and Rousk, 2010; Chaparro et al., 2012). In the present study, soil bacterial and fungal communities were significantly impacted by soil pH (Figure 5), which indicated that soil acidification rather than N availability shaped soil fungal and bacterial communities. Earlier studies have indicated a strong correlation between shifts in microbial community composition and soil pH (Francioli et al., 2016; Zhou et al., 2017; Zhang et al., 2021). Furthermore, soil acidification has been found to have a direct impact on the composition of soil microbial communities (Dai et al., 2019). However, on the other hand, it has been reported that N addition-induced changes in soil properties could exert positive (Zhou et al., 2017) and neutral (Turner and Joseph Wright, 2014) effects on soil microbial communities.

Interestingly, we found that there was a significant positive correlation between C-cycling enzyme activities and N-cycling enzyme activities (Figures 5A,B). This result is consistent with the research conducted by Dai et al. (2019), which demonstrated that C-cycling enzyme activity has significant relationship with the N-cycling enzymes. The observed phenomenon can be attributed to the impact of low soil C:N ratios on the activities of C-cycling enzymes. This leads to an increase in the degradation of cellulose, lignin, and other carbon sources that are typically challenging to decompose. As a result, there is a subsequent increase in the availability of C and the activities of N-related enzymes. Our study revealed that there was a significant positive relationship between soil pH and the activities of C cycling enzymes (Figure 5B). Enzymes are sensitive to changes in environmental conditions, including pH. Many C-cycling enzymes have an optimal pH range at which they function most efficiently. When the soil pH falls within the optimal range for these enzymes, their activity is enhanced, leading to increased decomposition and cycling of organic carbon compounds (Puissant et al., 2019). Soil pH can affect the solubility of minerals, which, in turn, can influence enzyme activity. Some minerals act as cofactors for C-cycling enzymes, and their availability might change with soil pH (Sheng et al., 2022). The results of this study are similar to those of Dai et al. (2019), who found a significant correlation between the activity of extracellular c-cycling enzymes and the pH of the soil.

It was further observed that across both soil types, fungal communities were significantly linked to C-cycling enzymes but not N-cycling enzymes (Figures 5A,B). Studies have claimed that soil fungal communities dominate SOC turnover and that fungi have a greater impact on soil carbon cycling than bacterial microbes in both acidic and basic soils (Xiao et al., 2018). The relationship between soil carbon cycling enzyme activities and soil fungi is critical for understanding carbon dynamics in terrestrial ecosystems. Soil carbon cycling enzyme activities are closely linked to the decomposition and transformation of organic matter, which affect the storage and release of carbon in the soil. Soil fungi play a key role in this process, as they are major contributors to organic matter decomposition and nutrient cycling in soils (Xiao et al., 2018). We further witnessed that C-cycling enzymes were highly linked to SOC (Figure 5A). SOC represents a reservoir of organic carbon in the soil. As the SOC content increases, it provides a continuous supply of carbon-rich substrates for microbial activity. Increased microbial activity, in turn, leads to higher enzyme production, facilitating further decomposition of organic matter and contributing to the maintenance of higher SOC levels. This creates a positive feedback loop where higher SOC leads to increased enzyme activity, which, in turn, supports the preservation and accumulation of more SOC (Wang et al., 2022).

## Conclusion

In conclusion, this research emphasizes the significant effects of N fertilization on soil properties, microbial communities, enzyme activities, and crop yield in fluvo-aquic and black soils in North China. Increased N fertilization led to improved nutrient availability, but at the same time, a higher N rate led to soil acidification across both soils. Moderate-N fertilization resulted in enhanced crop productivity over high N fertilization across both soil types. Microbial community composition and enzyme activities were influenced by N fertilization, with pronounced changes observed in the black soil. Soil pH was found to be a key driver in regulating soil microbial communities and enzyme activities. In addition, soil type resulted in the maximum variation in microbial communities and enzyme activities compared with N addition. Moreover, it is imperative for Chinese growers to transition from the practice of incorporating excessive quantities of fertilizer to employing minimal or optimal levels of fertilization. The implementation of this practice will not only enable growers to attain elevated crop yields but also avert the detrimental consequences of agriculture, specifically over-fertilization, on the ecosystem.

## Data availability statement

The raw data supporting the conclusions of this article will be made available by the authors, without undue reservation.

## Author contributions

SU: conceptualization, methodology, investigation, data curation, writingoriginal draft, and review and editing. MR, TA, and WZ: investigation, data curation, and review. XG: investigation and data curation. PH: conceptualization, funding acquisition, resources, and supervision. All authors reviewed and contributed to the article and approved the submitted version.

## Funding

This research was supported by Smart Fertilization Project, the National Key Research and Development Program of China (no. 2016YFD0200101), and CAAS-IPNI Joint Lab for Plant Nutrition Innovation Research.

## Conflict of interest

The authors declare that the research was conducted in the absence of any commercial or financial relationships that could be construed as a potential conflict of interest.

## Publisher’s note

All claims expressed in this article are solely those of the authors and do not necessarily represent those of their affiliated organizations, or those of the publisher, the editors and the reviewers. Any product that may be evaluated in this article, or claim that may be made by its manufacturer, is not guaranteed or endorsed by the publisher.
