# Supplementary material for: Responses of soil microbial communities and enzyme activities under nitrogen addition in fluvo-aquic and black soil of North China
Source: Front Microbiol. 2023 Aug 17;14:1249471. doi: 10.3389/fmicb.2023.1249471 (PMC10469899; doi:10.3389/fmicb.2023.1249471)
Supplement: Supplementary file 1 [file Data_Sheet_1.docx]

Supplementary Material

**Responses of soil microbial communities and enzyme activities under nitrogen addition in fluvo-aquic and black soil of north China**

**Sami Ullah^1,2^, Muhammad Mohsin Raza^3^, Tanveer Abbas^1^, Wei Zhou^1^, Ping He^1*^**

^1^Ministry of Agriculture Key Laboratory of Plant Nutrition and Fertilizer, Institute of Agricultural Resources and Regional Planning, Chinese Academy of Agricultural Science, Beijing 100081, PR China, ^2^ORIC, University of Baltistan, Skardu 16100, Pakistan, ^3^Soil Science Research Institute, National Agriculture Research Center, Pakistan Agricultural Research Council, Islamabad 44000, Pakistan

***Corresponding author:** heping02@caas.cn

# Table S1. The direct and indirect relationships between variables in fluvo-aquic soil. The path coefficients are calculated by PLS-PM after 1000 bootstrap.

| **S. No** | **Relationships** | **Direct** | **Indirect** | **Total** |
| --- | --- | --- | --- | --- |
| 1 | FERT > pH | -0.80 | 0.00 | -0.80 |
| 2 | FERT > N | 0.70 | 0.24 | 0.94 |
| 3 | FERT > SOC | 0.57 | 0.20 | 0.77 |
| 4 | pH > YIELD | 0.52 | -0.32 | 0.19 |
| 5 | pH > BACT | -0.97 | 0.07 | -0.90 |
| 6 | pH > FUNGI | -0.98 | 0.27 | -0.71 |
| 7 | pH > CE | -0.02 | -0.45 | -0.47 |
| 8 | pH > NE | -0.45 | -0.14 | -0.58 |
| 9 | N > YIELD | 0.98 | 0.00 | 0.98 |
| 10 | N > BACT | -0.34 | 0.00 | -0.34 |
| 11 | N > FUNGI | -1.00 | 0.00 | -1.00 |
| 12 | N > CE | 0.00 | -0.46 | -0.46 |
| 13 | N > NE | 0.29 | -0.21 | 0.09 |
| 14 | SOC > YIELD | 0.68 | 0.00 | 0.68 |
| 15 | SOC > BACT | 0.12 | 0.00 | 0.12 |
| 16 | SOC > FUNGI | 0.12 | 0.00 | 0.12 |
| 17 | SOC > CE | 0.87 | 0.03 | 0.90 |
| 18 | SOC > NE | 0.00 | 0.38 | 0.38 |
| 19 | BACT > CE | -0.32 | 0.00 | -0.32 |
| 20 | BACT > NE | -0.26 | -0.14 | -0.41 |
| 21 | FUNGI > CE | 0.57 | 0.00 | 0.57 |
| 22 | FUNGI > NE | 0.09 | 0.25 | 0.35 |
| 23 | CE > NE | 0.45 | 0.00 | 0.45 |

Table S2. The direct and indirect relationships between variables in black soil. The path coefficients are calculated by PLS-PM after 1000 bootstrap.

| **S. No** | **Relationships** | **Direct** | **Indirect** | **Total** |
| --- | --- | --- | --- | --- |
| 1 | FERT > pH | -0.65 | 0.00 | -0.65 |
| 2 | FERT > N | 0.60 | 0.03 | 0.63 |
| 3 | FERT > SOC | 0.64 | -0.18 | 0.46 |
| 4 | pH > YIELD | -0.53 | 0.12 | -0.40 |
| 5 | pH > BACT | 0.35 | -0.58 | -0.23 |
| 6 | pH > FUNGI | -0.86 | 0.26 | -0.60 |
| 7 | pH > CE | 0.62 | 0.17 | 0.79 |
| 8 | pH > NE | -0.18 | 1.00 | 0.82 |
| 9 | N > YIELD | 0.71 | 0.00 | 0.71 |
| 10 | N > BACT | 0.58 | 0.00 | 0.58 |
| 11 | N > FUNGI | 0.24 | 0.00 | 0.24 |
| 12 | N > CE | 0.00 | 0.13 | 0.13 |
| 13 | N > NE | -0.29 | 0.04 | -0.25 |
| 14 | SOC > YIELD | 0.43 | 0.00 | 0.43 |
| 15 | SOC > BACT | -0.28 | 0.00 | -0.28 |
| 16 | SOC > FUNGI | -0.19 | 0.00 | -0.19 |
| 17 | SOC > CE | 0.18 | -0.08 | 0.10 |
| 18 | SOC > NE | 0.00 | 0.13 | 0.13 |
| 19 | BACT > CE | 0.13 | 0.00 | 0.13 |
| 20 | BACT > NE | -0.15 | 0.12 | -0.03 |
| 21 | FUNGI > CE | 0.74 | 0.00 | 0.74 |
| 22 | FUNGI > NE | 0.00 | 0.22 | 0.22 |
| 23 | CE > NE | 0.91 | 0.00 | 0.91 |
