# Supplementary material for: Responses of soil microbial communities and enzyme activities under nitrogen addition in fluvo-aquic and black soil of North China
Source: Front Microbiol. 2023 Aug 17;14:1249471. doi: 10.3389/fmicb.2023.1249471 (PMC10469899; doi:10.3389/fmicb.2023.1249471)
Supplement: Supplementary file 2 [file Data_Sheet_2.docx]

**Figure 1**

Principle component analysis (PCA) of the soil microbial community composition (A) and extracellular enzyme activities (B) data for the control, moderate-N, and high-N treatments for fluvo-aquic and black soil.

**Figure 2**

Permutational multivariate analysis of variance (PERMANOVA) comparing the main and interactive effects of N fertilizer and soil type on the soil microbial community composition (A) and extracellular enzyme activities (B) at (999 permutations). Asterisks indicate significant differences at *P ≤ 0.01 and **P ≤ 0.001 probability levels.

**Figure 3**

Significantly altered N and P cycling extracellular enzyme activities in fluvo-aquic and black soil.

Different letters in a column indicate significant differences among treatments at P ≤ 0.05 probability level as determined by Fisher’s least significant difference (LSD). The data are the means; n = 4.

Fluvo-aquic soil: control = no N applied; moderate-N = N182 kg ha^-1^; high-N = N225 kg ha^-1^

Black soil: control = no N applied; moderate-N = N200 kg ha^-1^; high-N = N251 kg ha^-1^

**Figure 4**

Significantly altered N and P cycling extracellular enzyme activities in fluvo-aquic and black soil. Different letters in a column indicate significant differences among treatments at P ≤ 0.05 probability level as determined by Fisher’s least significant difference (LSD). The data are the means; n = 4.

Fluvo-aquic soil: control = no N applied; moderate-N = N182 kg ha^-1^; high-N = N225 kg ha^-1^

Black soil: control = no N applied; moderate-N = N200 kg ha^-1^; high-N = N251 kg ha^-1^

**Figure 5**

Directed graph of the partial least squares path model (PLS-PM) for fluvo-aquic soil (A) and black soil (B). Each box represents an observed variable (i.e., measured) or latent variable (i.e., constructs). The loading for N contents and the six enzyme activities that create the latent variables are shown in the dashed rectangle. Path coefficients are calculated after 1000 bootstraps and reflected in the width of the arrow, with blue and red indicating positive and negative effects, respectively. Dashed arrows show that coefficients did not differ significantly from 0 (P ≥ 0.05). The model is assessed using the Goodness of Fit (GoF) statistic, and the GoF values were **0.70 and 0.66** in the fluvo-aquic and black soil, respectively.
